# Digital Prompts to Increase Engagement With the Headspace App and for Stress Regulation Among Parents: Feasibility Study

**DOI:** 10.2196/30606

**Published:** 2022-03-21

**Authors:** Lisa Militello, Michael Sobolev, Fabian Okeke, Daniel A Adler, Inbal Nahum-Shani

**Affiliations:** 1 College of Nursing The Ohio State University Columbus, OH United States; 2 Cornell Tech Cornell University New York, NY United States; 3 Feinstein Institute for Medical Research Northwell Health Great Neck, NY United States; 4 Institute for Social Research University of Michigan Ann Arbor, MI United States

**Keywords:** Headspace, engagement, mHealth, mindfulness, mental health, mobile phone

## Abstract

**Background:**

Given the interrelated health of children and parents, strategies to promote stress regulation are critically important in the family context. However, the uptake of preventive mental health is limited among parents owing to competing family demands.

**Objective:**

In this study, we aim to determine whether it is feasible and acceptable to randomize digital prompts designed to engage parents in real-time brief mindfulness activities guided by a commercially available app.

**Methods:**

We conducted a 30-day pilot microrandomized trial among a sample of parents who used Android smartphones. Each day during a parent-specified time frame, participants had a 50% probability of receiving a prompt with a message encouraging them to engage in a mindfulness activity using a commercial app, Headspace. In the 24 hours following randomization, ecological momentary assessments and passively collected smartphone data were used to assess proximal engagement (yes or no) with the app and any mindfulness activity (with or without the app). These data were combined with baseline and exit surveys to determine feasibility and acceptability.

**Results:**

Over 4 months, 83 interested parents were screened, 48 were eligible, 16 were enrolled, and 10 were successfully onboarded. Reasons for nonparticipation included technology barriers, privacy concerns, time constraints, or change of mind. In total, 80% (8/10) of parents who onboarded successfully completed all aspects of the intervention. While it is feasible to randomize prompt delivery, only 60% (6/10) of parents reported that the timing of prompts was helpful despite having control over the delivery window. Across the study period, we observed higher self-reported engagement with Headspace on days with prompts (31/62, 50% of days), as opposed to days without prompts (33/103, 32% of days). This pattern was consistent for most participants in this study (7/8, 87%). The time spent using the app on days with prompts (mean 566, SD 378 seconds) was descriptively higher than on days without prompts (mean 225, SD 276 seconds). App usage was highest during the first week and declined over each of the remaining 3 weeks. However, self-reported engagement in mindfulness activities without the app increased over time. Self-reported engagement with any mindfulness activity was similar on days with (40/62, 65% of days) and without (65/103, 63% of days) prompts. Participants found the Headspace app helpful (10/10, 100%) and would recommend the program to others (9/10, 90%).

**Conclusions:**

Preliminary findings suggest that parents are receptive to using mindfulness apps to support stress management, and prompts are likely to increase engagement with the app. However, we identified several implementation challenges in the current trial, specifically a need to optimize prompt timing and frequency as a strategy to engage users in preventive digital mental health.

## Introduction

### Background

The stressors of parenting are normative and unavoidable. Broadly, stress is viewed in the context of life events (major or minor) that disrupt mechanisms intended to maintain one’s physiology, emotion, and cognition [1]. Specifically, parenting stress is a natural experience that arises when parenting demands exceed expected and available resources [2]. Parenting stress may reflect broader contexts to include any number of major life stressors, relationships, or family circumstances [3,4]. Parenting stress can have a negative effect on relationship quality, health, mood, and overall well-being of not only the parent but also the family [3,5,6]. In both children and adults, acute and chronic stress are linked with numerous physiological and psychological disease states [5,7-9]. Thus, strategies to regulate stress are critically important among children and families [2,5-7].

However, few parents seek professional help to manage stress, and few primary care providers counsel on stress management skills [10]. Before the COVID-19 pandemic, 2019 data show that only 9.5% of adults aged ≥18 years reported receiving mental health counseling in the past year [11]. The COVID-19 pandemic took a particularly heavy toll on parents with children aged ≤18 years without much support. Evidence shows that stress management counseling is rarely offered in primary care or is the least common type of counseling provided by primary care physicians relative to diet, physical activity, or smoking cessation [10,12].

Stress management exercises (eg, mindfulness, cognitive reframing, and behavioral modification) are empirically supported strategies to promote self-regulatory skills, stress reduction, and resilience [13-15]. Evidence suggests that mindfulness interventions have the potential to reduce parenting stress and improve psychological functioning in youth [5]. Specifically, mindful parenting (ie, moment-to-moment awareness of the parent-child relationship) may reduce parental stress and promote family well-being [6,16]. However, it is unclear how formal (ie, purposeful or dedicated time) or informal (ie, weaving mindfulness into existing routines such as dishwashing) mindfulness is required to yield positive outcomes [17,18]. Mindfulness intervention dosage has wide variability reported anywhere from 9 to 27 hours [5], 45 minutes to 2 hours delivered over 3 to 12 sessions [19], and 4.5 to 24 hours [20]. Collective findings from systematic reviews and meta-analyses suggest small but beneficial effects of mindfulness interventions [5,19,20].

However, American families are often busy citing time and logistical barriers (costs and transportation) as reasons for attrition or nonparticipation in health-promoting activities. Both before and during the COVID-19 pandemic, families struggled to balance work and life, often feeling tired, rushed, and short on quality time with their children, friends, and hobbies [21]. In 2019, around 51% of mothers and 82% of fathers reported working full-time. In 2015, 26% of children aged <18 years lived with a single parent, and 53% to 73% of parents reported that their child participated in an extracurricular activity in the previous 12 months [21].

Brief mindfulness interventions may be a suitable alternative to more traditional mindfulness programs for populations with limited time or available resources [22]. In contrast to traditional 8- to 10-week mindfulness interventions, brief mindfulness interventions range from 3- to 5-minute guided exercises to 2-week programs [22]. Brief mindfulness interventions have also become increasingly popular with the rise of commercially available mindfulness meditation apps and other technologies [22-25]. Both commercial and research-developed mobile apps that help participants learn, practice, and monitor mindfulness activities [17,18,25,26] may increase opportunities to learn and practice mindfulness or meditation (hereinafter collectively referred to as mindfulness) across diverse audiences compared with traditional face-to-face programs [27], and successful coping strategies may be deployed in real time in real-world conditions to mitigate the deleterious effects of parenting stress [2,5,28].

A critical challenge in digital health, the law of attrition, occurs when digital health study participants drop out before completion or stop using the app [29,30]. Interventions intended to revolutionize stress regulation, including digitally supported health interventions, often fail because they are not used in real-world settings [31-35]. The main users of mental health apps are predominantly younger individuals, of high socioeconomic status, have overall positive health, and routinely engage in preventive health practices [36-39]. Families, particularly of low income, have poor adoption rates of abundant mobile health (mHealth) solutions despite high smartphone ownership and potential for scalability [32,33,38]. Poor engagement with digital health interventions may be mitigated through real-world strategies [31,40,41]. Many mHealth systems lack sufficient effort devoted to their design, development, and evaluation across diverse populations [15,31,34]. A solution-focused approach prioritizes the development of a solution to a practical problem to produce a sustainable solution [40,42].

A critical first step in supporting parental stress regulation (distal outcome) was to identify whether and under what conditions prompting parents to engage in mindfulness is beneficial. Digital prompts (eg, push notifications) are frequently used to promote engagement. Digital prompts are intended to nudge users in a particular direction without limiting the freedom of choice [43-45]. However, an overflow of notifications can be burdensome, leading users to ignore push notifications, increase user inattention, and/or exacerbate disengagement from apps [30,46]. Digital prompts are subject to a myriad of factors such as timing, frequency, sender, content, message framing, mode of delivery, and theoretical underpinning [43-45]. Previous research on the use of digital health prompts to increase engagement with interventions among parent populations is limited [47,48]. One study conducted with parents found that the timing of prompts to support healthy lifestyle behaviors may be beneficial if delivery coincides with parents’ perceived need for support (eg, prompt to practice guided imagery sent when the parent was at home versus while driving) [47]. Parent preference for prompt timing (ie, self-selected time frame of when to receive a prompt) peaked during late afternoon and evening hours when the school or workday ended and the family transitioned to dinnertime and evening activities [47]. With regard to prompt frequency, survey data show that parents desire few notifications (ie, 2 times per week) [48], while intervention data suggest that parents favor frequent messages (eg, daily) [47,48]. As mobile apps evolve, opportunities for meaningful intervention within life patterns may be possible through various mechanisms that sense and capture streams of personal data, giving insight into contextual factors [43,44,49-51].

Just-in-time adaptive interventions (JITAIs) have potential for shaping health behavior, using various data streams to deliver prompts at the *right time* while minimizing user burden and habituation [52]. JITAIs rely on explicit decision rules for when to prompt users with specific intervention components [52]. However, it is uncertain when and under what conditions, interventions should be delivered to engage parents in real-time stress-regulating activities. A microrandomized trial (MRT) is an experimental design that can supplement the use of theory to guide JITAI development. An MRT allows researchers to study the proximal effects of a specific intervention component, change over time, and contextual factors that may moderate time-varying effects [52].

Therefore, before designing a comprehensive JITAI for parenting stress, we conducted a pilot MRT to explore whether it was possible to leverage digital prompts to support real-time parent engagement with stress-regulating activities guided by a commercially available mindfulness app, Headspace. We developed a novel system that, from the front end (parent perspective), leveraged digital prompts containing messages that encouraged brief (<10 minutes) mindfulness activities and were capable of launching Headspace. Parents were able to change the timing of prompt delivery to suit their individual needs through a secondary research-developed app. From the back end (researcher perspective), the system would randomize to send or not send a digital prompt during the parent-specified window. Headspace was used to teach and support mindfulness activities. While adaptability and scalability were facilitated using a commercially available app, proprietary back-end data for Headspace were not accessible by our research team (ie, what content parents accessed on Headspace). Therefore, our system was designed to passively capture relevant smartphone data (eg, when the Headspace app was open; how long the app was open; or smartphone paradata such as silent, ring, or vibrate mode). Finally, our system pushed a daily ecological momentary assessment (EMA). The EMA was used to differentiate engagement with the app versus engagement with mindfulness and to observe for potential benefits on parent mood.

### Objective

In this study, we aim to conduct a pilot MRT to determine if it was feasible and acceptable to randomize digital prompts designed to engage parents in real-time brief mindfulness activities guided by a commercially available app (Headspace).

## Methods

### Trial Design

**Figure 1:**
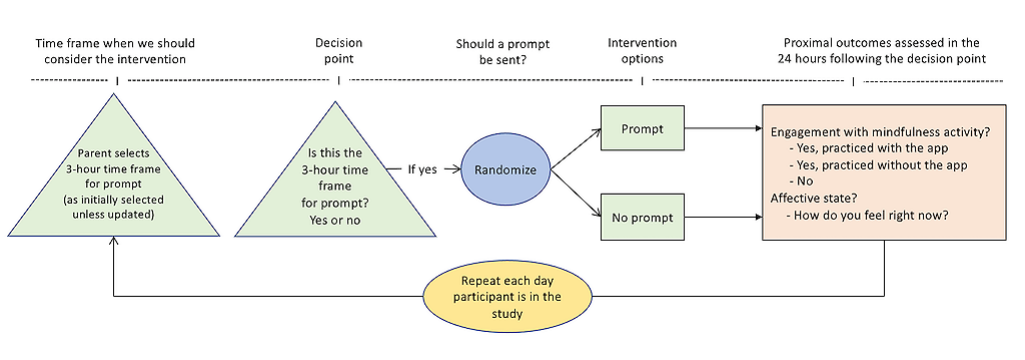
Trial design.

We conducted a 30-day feasibility and acceptability study whereby participants were microrandomized [53] once daily with equal probability to either receive or not receive a prompt recommending engagement in a commercially available mindfulness app (Figure 1). Microrandomizations took place within a time window that each participant prespecified as convenient to receive the prompt. Specifically, during the onboarding session, participants were asked to specify a 3-hour window during the day to receive a prompt encouraging them to practice mindfulness. On the basis of our prior work [47], a 3-hour window was thought to be broad enough but not so restrictive to capture windows of family routines (eg, morning before work or school, work or school lunch or breaks, after work or school dinner, evening activities, or bedtime routines). The time frame can be updated at any point by the user. If a prompt was sent, the participant could either tap the prompt and launch a mindfulness app or dismiss the prompt. Prompt messages were neutral in tone and picked at random from a library developed for the study (eg, “Take your mind to your calm place”; “Use mindfulness to develop your mental toughness”; “10 minutes for yourself can make a huge difference”; and “Give yourself time to recharge”). In the 24 hours following randomization, proximal outcomes of engagement in mindfulness exercises and affect were self-reported via daily EMAs and passively assessed via the smartphone.

### Procedure and Ethics Approval

From November 2018 to February 2019, participants were recruited from web-based or social media announcements, word of mouth, and targeted community and workplace recruitment (eg, email notifications via listservs). Announcements included information regarding the study, contact information, and a link to a 3-item eligibility screening survey. Owing to technical aspects of the platform, participant inclusion criteria were as follows: (1) self-identified parent with child or children (aged up to 18 years) at home and (2) an Android smartphone user. The exclusion criterion was non–English-speaking participants. Upon meeting the inclusion criteria, participants provided written informed consent and were enrolled in the study. The setting was the real-world, everyday lives of the participants. Given the formative nature of the proposed work, a convenience sample was used. In appreciation of participant time and feedback, parents were compensated commensurate with participation. Parents were provided compensation for baseline survey completion (US $5), onboarding (US $5), EMA (up to US $15), exit survey (US $5), and a 1-month Headspace Plus paid subscription (valued at approximately US $13). The lead institution was the Ohio State Behavioral and Social Sciences Institutional Review Board (IRB) with ethical approval from the Ohio State University Office of Responsible Research Practices (IRB #2017B0550).

**Figure 2:**
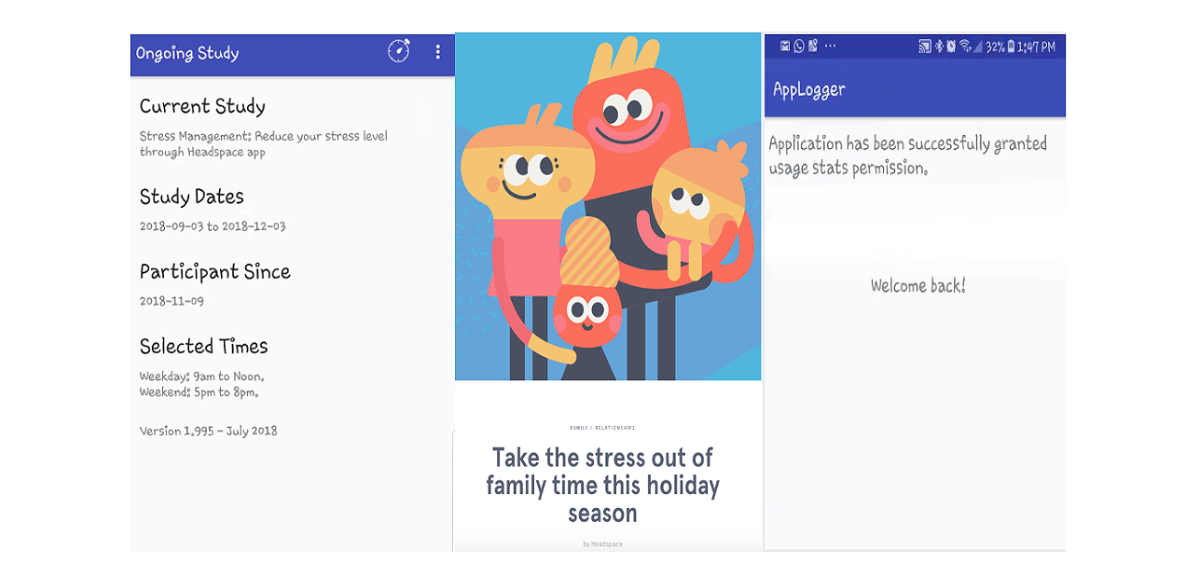
Research apps Beehive, HeadspaceTM, and App Logger.

After obtaining informed consent and completing baseline surveys, participants were provided with onboarding instructions. As part of feasibility testing and formative evaluation, participants were asked to install 3 mobile apps necessary to conduct the research (Figure 2). Two research-developed apps (Beehive and App Logger) were made available to participants through a provided link, while one app (Headspace) was commercially available for download through the Google Play Store. It is worth noting that the research team submitted a request for collaboration with Headspace but owing to timing and resource limitations, the Headspace Health Partnership team was unable to accept our request. However, the principal investigator (PI) communicated with a member of the Headspace team to discuss logistical questions related to onboarding participants. Upon study completion, participants were asked to complete exit surveys and uninstall the research-affiliated apps. Given the commercial availability of Headspace, this app could be continued at the user’s discretion.

### Mobile Apps

#### Beehive

Beehive is a research-developed app that participants installed to guide the delivery of study prompts [54] and the EMA questions. Participants used Beehive to indicate daily wake and bedtime schedules and allowed participants to select the preferred timing for the digital prompts. Specifically, participants were instructed to use the Beehive app to select a 3-hour window to potentially receive intervention prompts encouraging them to practice mindfulness. Parents were informed that they could update the 3-hour window through the Beehive app at any point during the study. The Beehive app also delivered the EMA question at the end of the day and at least one hour before the specified bedtime of each participant.

#### Headspace App

Prior research has leveraged commercially available tools to support stress regulation and optimize digital health interventions [26,44,55]. We selected Headspace, a commercially available mobile app that includes a wide collection of mindfulness exercises that vary in terms of length and topic [56]. In a review of mindfulness-based iPhone apps and using the Mobile Application Rating Scale [55] to determine app quality, Mani et al [24] found Headspace to have the highest average score. Participants were provided with a code that gave them access to the full Headspace library and were asked to use the app over the course of 1 month. Headspace includes push notifications that participants were asked to disable during the study.

#### App Logger

App Logger is a research-developed app used to facilitate data collection. It is an unobtrusive mobile app for Android devices that passively records and timestamps smartphone paradata [54]. App Logger was used in prior studies to objectively measure app usage but not content [54,57]. It was used to passively capture smartphone paradata, such as when an app was launched, duration of use (removed from the foreground), and smartphone mode (eg, locked or unlocked). In lieu of a partnership with Headspace, App Logger was used to passively collect relevant smartphone paradata and contribute to a holistic view of engagement.

### Measures

#### Feasibility and Acceptability

Feasibility and acceptability were assessed via participant enrollment and retention rates, satisfaction and acceptability ratings (a benchmark of ≥90%), estimates of use, self-reported engagement with mindfulness exercises (via EMA) and objective engagement with the app (ie, passively collected app usage, patterns, and trends over time), reactions to the intervention, factors affecting implementation ease or difficulty, and the ability of participants to carry out study activities [58]. A research log was used to document the proportion of eligible parents, relative to those who enrolled and subsequently those who enrolled compared with actual attendance. These data were used to inform whether we were able to recruit our target population. We determined whether randomization was feasible by monitoring software performance and was acceptable by monitoring for aberrant data. Retention rates and reasons for nonparticipation were obtained when possible. A database of prompts sent, date, and timestamps was maintained. Additional smartphone paradata, information regarding smartphone use, were assessed using the App Logger app. App Logger passively records when a prompt is delivered, time from delivery to launching the Headspace app, and duration the Headspace app is open (not content). In addition, App Logger can detect the smartphone mode and setting (eg, on or off or silent, vibrate, or ring mode). We have reported on all study outcomes to determine the extent and likelihood that the intervention was implemented as planned.

#### Baseline Survey

The baseline survey collected basic demographic information and previous experiences with mindfulness. In addition, we assessed for the personality factor, neuroticism, using the neuroticism subscale of the Big Five Personality Inventory [59,60]. Neuroticism is an indicator of emotional stability, with higher scores suggesting a higher likelihood of vulnerability to stress, anxiety, and nonspecific mental distress [61-63]. The neuroticism subscale consists of 8 items rated on a 5-point scale where 1=disagree, 3=neutral, and 5=agree. To assess depression, anxiety, and stress, we used the Depression Anxiety Stress Scale 21-item (DASS-21) [64]. The DASS-21 has been successfully used in a variety of clinical and nonclinical settings, including parent populations [65-67]. Participants answered each item based on how it applied to them in the past week. Responses ranged from 0=did not apply to me at all to 3=applied to me most of the time. The DASS-21 cutoff values indicate levels such as normal, mild, moderate, severe, and extremely severe within each state; that is, depression, anxiety, or stress.

#### Ecological Momentary Assessments

Nightly EMAs were scheduled for delivery within 1 hour of participant-indicated bedtimes. We used the Photographic Affect Meter (PAM [68]) to measure the daily affective state. The PAM typically takes users less than a minute to complete, asking users to choose from a grid of 16 photographs arranged in a 4 × 4 grid that most represents their current state. Each image was mapped to the established valence and arousal states. Participants were asked to “touch the photo that best captures how you feel right now.” The output of this selection is a positive or negative affect value, which has been validated in multiple trials and shown to be an effective alternative to longer-form surveys [68,69].

Self-reported engagement in mindfulness exercises was measured by asking participants whether they had performed a mindfulness activity. Response options were “Yes, I used the app,” “Yes, I practiced on my own,” or “No.” These options were provided to ascertain how participants engaged in mindfulness exercises, with versus without the app.

#### End-of-Study Survey

A 14-item survey consisting of both open- and close-ended questions was used to elicit participants’ overall thoughts about the study, prompt-specific feedback, and Headspace-specific feedback (ie, prompt delivery, prompt content, and Headspace app). Survey questions were about general perceived ease of use, usefulness, and satisfaction based on input from prior work with parent populations [47,48], usability [70], and expert recommendations for feasibility and acceptability studies [58,71].

Examples of yes or no questions included the following: (1) Was it easy to get set up and started? (2) Did you dismiss any notifications? (3) Did you find the overall program useful in managing your stress? (4) Would you recommend the program to other parents? Prompt-specific questions included questions such as the following: (1) Was the number of messages sent (frequency) too much, too little, or just right? (2) Was the time when the prompt was delivered helpful, not helpful, or just right? (3) Did you like the wording of the messages: yes or no? (4) Did your feelings toward the notifications change the longer you were in the study? (5) Was there anything that we could have done better? Headspace-specific questions included the following: (1) Did you find the Headspace activities helpful in managing your stress? (2) Did you like the graphics and characters used in Headspace? (3) Can you list one Headspace activity that you liked most and least? (4) Do you think it would be helpful for your kids to use Headspace to learn mindfulness?

### Data Evaluation

Feasibility and acceptability were assessed via participant enrollment and retention rates, satisfaction, factors affecting implementation ease and difficulty, patterns of use, and ability of participants to carry out study activities [58]. The proximal outcome, engagement with a mindfulness activity, was assessed in the 24 hours following randomization (when the system would randomize to send or not send a prompt). Engagement in a mindfulness activity was operationalized to disentangle engagement with the app (eg, simply opening the Headspace app) versus engagement with a mindfulness activity (eg, deep breathing with the app or deep breathing on my own without the app). Engagement in any mindfulness activity was assessed along with the affective state (transient emotions) from daily EMAs.

Quantitative data were analyzed descriptively. Benchmarks for recruitment and enrollment efforts were set at ≥50 participants screened and 20 participants enrolled (most studies had a sample of <50 participants; nearly one-third—ie, 6/2, 30%—of studies had a sample of <20 participants) based on a systematic review of similar research [20] and given the pilot nature of the proposed work. Additional indicators of recruitment success were if ≥24% of the enrolled participants represented racial or ethnic minorities [20]. Owing to limited supporting evidence, we also reported on frequencies of participation from low-income families and fathers (compared with mothers) to help fill the gaps in knowledge. We anticipated 100% success in onboarding and aimed for <20% attrition during the 30-day intervention implementation. We report on response rates to EMAs as we have done in our prior work, with a benchmark for success calculated at 60% or higher (response rate 49% for 35 days [47] and 61% for 30 days [72]).

Qualitative data from free-response questions was narratively summarized by 2 coders representing 2 different disciplines. The study PI (LM) served as the lead coder based on the population and subject matter expertise. The second coder (MS) provided topical expertise on the subject and helped to verify the findings against actual data. Any areas of concern were discussed. The process began with an initial reading of the data before coding. Multiple readings of free-response data were performed to identify key words or phrases. Passages coded similarly were grouped into themes to provide a more comprehensive view of the data.

## Results

### Sample

**Figure 3:**
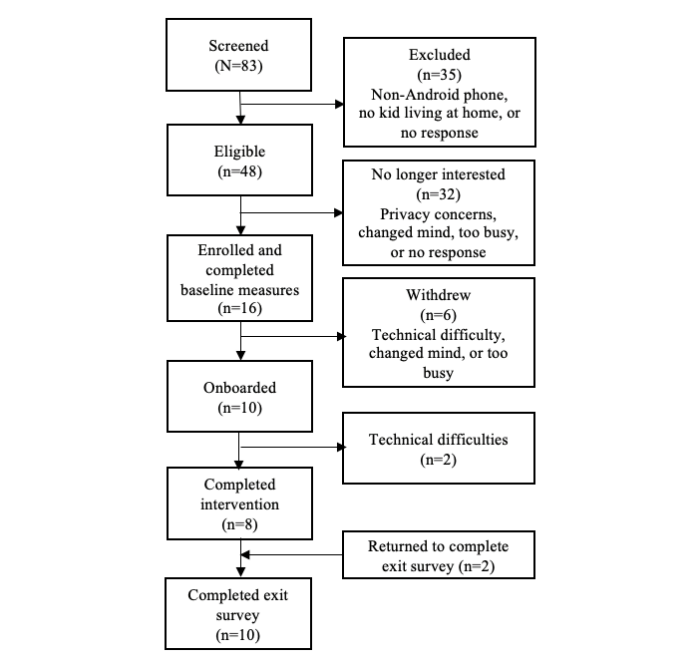
CONSORT (Consolidated Standards of Reporting Trials) diagram.

Over the course of 4 months, we screened 83 parents who expressed interest in the research. Approximately 42% (35/83) of the parents were excluded before conducting the research, mainly because they were not Android users. Of the 48 eligible patients, 16 (33%) parents were enrolled. Figure 3, the CONSORT diagram, depicts enrollment, participation, and analysis. Most of our sample identified as White and female and were aged between 35 and 44 years. All participants used Android devices as their primary mobile smartphone, with Samsung being the predominantly reported brand (6/10, 60%). At baseline, the sample average score for neuroticism was moderate (approximately 26 out of 40), suggesting a sample not particularly calm nor prone to emotional instability. Similarly, assessments of depression, anxiety, and stress were largely within normal ranges. Although most of the participants reported practicing stress management (13/16, 81%), fewer reported using mindfulness (6/16, 38%) or the Headspace app (1/16, 6%). Table S1 in Multimedia Appendix 1 shows the sample characteristics.

### Feasibility

Recruitment and enrollment were conducted on a rolling basis, but intervention implementation was grouped into waves to prevent overlap with the winter holiday season and to meet logistical needs associated with deploying Headspace bulk subscriptions. As such, most participants were screened and enrolled during the busy winter holiday season (November-December: 12/16, 75%), while efforts slowed after the New Year (January-February: 4/16, 25%). In addition, 12% (2/16) were fathers, 12% (2/16) reported receiving medical assistance, and 6% (1/16) represented a racial or ethnic minority. We were able to meet our recruitment goal but were shy of our enrollment goal and unable to meet our goal to have ≥24% of racial or ethnic minority participation. Enrollment was further aggravated with reductions in sample size occurring during the onboarding process.

Collectively, 50% (8/16) of parents reported technical difficulties during the onboarding process, but 25% (2/8) were able to overcome these barriers after speaking with a member of the research team. In wave 1, 33% (4/12) of participants were lost during onboarding, and in wave 2, a total of 50% (2/4) of participants were lost during onboarding. Logistical challenges during onboarding can be broadly categorized into two categories: (1) integrating a commercial app into research and (2) using research-developed apps in real-world settings. First, it was necessary for the PI to collaborate with the Headspace team to navigate the logistics of how to purchase and transfer separate Headspace subscriptions to each participant. At the time, Headspace allowed for bulk subscription purchases, akin to a corporate account, but required at least 30 subscriptions to be purchased at once. Furthermore, bulk subscriptions had to be activated on the same day, which was counter to the rolling recruitment research protocol. This was solved in 2 ways. We arranged a small cohort of participants to start the study on the same day using a promo code associated with a bulk subscription. In addition, we provided individual Headspace gift subscriptions to participants via rolling recruitment. Both strategies allowed parents to download the Headspace app from the Google Play Store and enter a promo code to unlock the premium version of Headspace. Most parents were familiar with the Google Play Store and located the Headspace app for download. In total, 3 of 16 (19%) parents reported that the Headspace promo code did not work when they were onboarding. During these scenarios, the PI helped troubleshoot to resolve the issue. The second challenge was the implementation of the 2 research apps, both available via GitHub. Participants were provided with a direct link to download the research apps. Although the research apps allowed the research team to have greater control, some smartphones triggered a warning during installation. Such warnings may be standard on some Android devices when an app is downloaded from a location other than the Google Play Store. However, parents reported being unfamiliar with downloading apps from outside the Google Play Store:

I can’t download apps from an outside source that isn’t Google Play. Unfortunately it looks like I won’t be able to participate in the study.

P5

Similarly, parents voiced concerns regarding privacy and smartphone access, although they were informed that the content was not being monitored:

I’m fine with it tracking Headspace usage, but I’m a bit concerned if it’s monitoring all my app, location, calls or/and keyboard usage.

P7

It’s like asking someone to put a chair in your living room, even more so it’s like asking someone to move in, especially when they are requesting access on your phone.

P4

**Figure 4:**
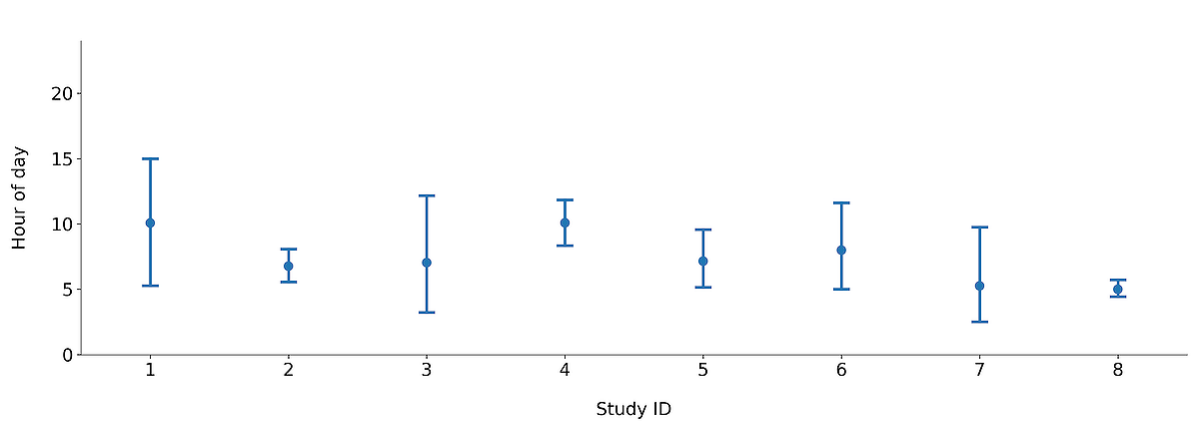
Distribution of hour of day (from 0 to 24, after midnight) when a participant opened the Headspace app.

Ultimately, 63% (10/16) of the enrolled parents onboarded to the 30-day intervention phase of the study. However, incoming data streams from App Logger were interrupted for 2 of 10 (20%) participants. We were unable to ascertain with certainty whether these interruptions were due to system issues or whether these participants chose to alter or revoke App Logger permissions. Thus, 8 of 10 (80%) parents fully completed the intervention phase and provided the following insights. An average of 10 (SD 5.44) prompts per participant was delivered over the 30 days relative to intended on-average 15 prompts (ie, 50% chance of receiving a prompt × 30 days in the study). Prompt messaging may be found in Multimedia Appendix 2. Across the 8 parents, smartphones were set to the normal ring mode 46% of the time when a prompt was sent (44 observed instances where the smartphone was set to the normal ring mode/95 prompts sent). The average time from prompt delivery to action (eg, either launch Headspace or dismiss notifications) was 185 (SD 303) minutes. A reliable reference point for nonprompt to action was not available; therefore, a comparison analysis was not possible. Figure 4 shows the hour of day (from 0 to 24 hours, after midnight) when a participant opened the Headspace app. The x-axis separates participants, shows the mean as the point, and shows the 95% CI around the mean as error bars. From the 95% CIs, we see visually that the variability in the time of day of opening Headspace differed, with some individuals using Headspace at a more consistent time of day than others.

Proportionally, most prompts were dismissed if the smartphone was in the silent mode (Table 1). The response rate to EMA surveys was 72.4% (ie, 165 EMA responses/228 EMAs pushed).

**Table 1:** Smartphone ringer mode when the prompt was sent.

Ringer mode	Value (n=82), n (%)	Was the prompt dismissed?^a^	
		Yes (by mode), n (%)	No (by mode), n (%)
Normal	39 (48)	22 (56)	17 (44)
Silent	12 (15)	9 (75)	3 (25)
Vibrate	31 (38)	18 (58)	13 (42)

^a^The “Value” column was used as the denominator.

#### Engagement

##### Engagement With the Headspace App

**Figure 5:**
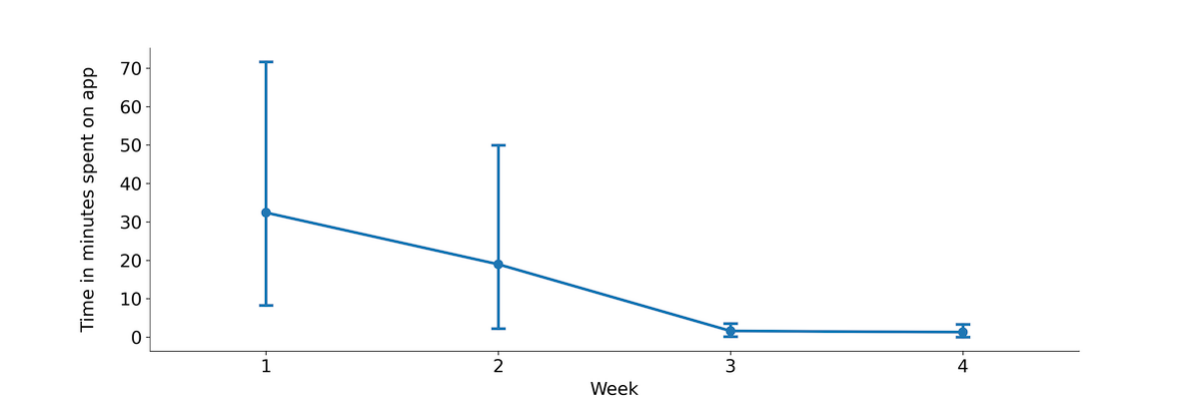
Total duration of participant engagement with app by week in study across all participants (n=8). Points represent the mean value, and error bars around each point represent a 95% CI around the mean.

Over 30 days and across the 8 participants, we counted 298 engagements with the Headspace app from passively collected smartphone data. We noted higher self-reported engagement with the app on days when a prompt was sent (31/62, 50% of days) compared with days without a prompt (33/103, 32% of days). Across the study, most participants (7/8, 87%) in the study self-reported higher engagement with the app on days with prompts. Engagement with the app was also found to be longer on days with the prompt (mean 566, SD 378 seconds) than on days without a prompt (mean 225, SD 276 seconds). Across the 8 participants, we observed that most engagements with the Headspace app occurred during the first week of the study but subsequently tapered down by week 4. Figure 5 highlights the total duration of engagement with the app by week in the study across the entire sample. The high error bars in weeks 1 and 2 suggest a larger variation in individual use during the first 2 weeks compared with the last 2 weeks.

The median (IQR) number of engagements with the app per user was 19 (10-55). The median (IQR) duration of each log-in was 45 (15-110) seconds. We discovered 25 discrepancies between reported (yes or no) and observed (yes or no) app usage, where parent-reported data from EMAs indicated app usage, yet we found no recorded Headspace app use on passively recorded data. On exit surveys, 50% (5/10) of parents reported using the app for an average of 5 to 10 minutes per day, and 40% (4/10) reported using the app 1 to 3 days per week.

##### Self-reported Engagement With Mindfulness Exercises (Based on Daily EMAs)

**Figure 6:**
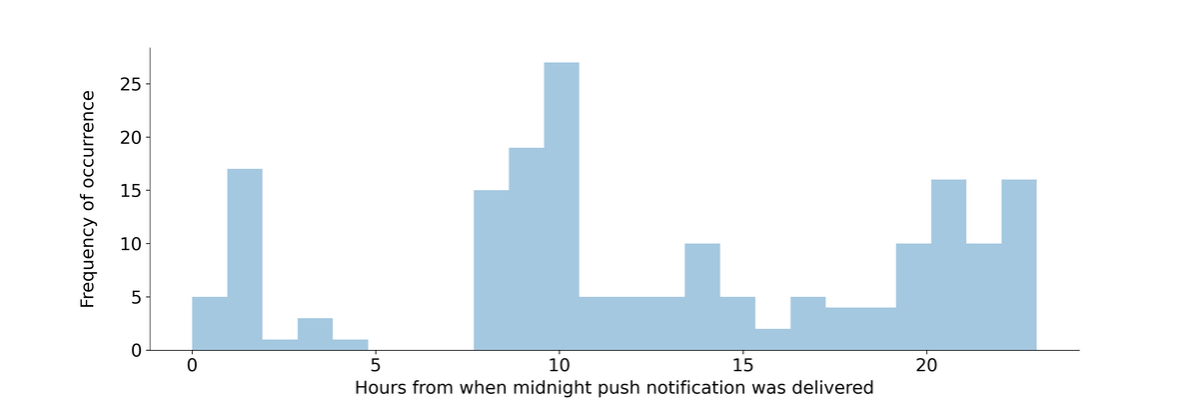
Hours from midnight prompts were sent based on the parent-selected time frame. Each bar represents a single hour of the day, and the height of the bars represents the total number of push notifications sent at that specific hour across all participants in the study.

On the basis of when prompts were sent, we observed a multimodal distribution for the time of day when the prompt was sent, most often during the morning hours, peaking between 9 AM and 10 AM (Figure 6). Proportionally, parent-reported engagement in a mindfulness activity (Table 2) was descriptively similar on days when a prompt was not sent (65/103, 63%) compared with that on days when a prompt was sent (40/62, 65%).

Collectively, parents reported using the Headspace app for mindfulness activity (64/105, 61%) more than engaging in mindfulness activities without the app (41/105, 39%; Table 2).

**Figure 7:**
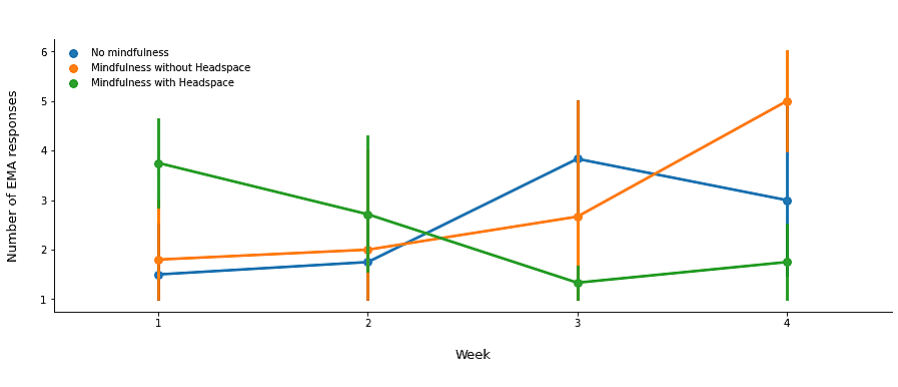
Self-reported engagement with a mindfulness activity by week in study (n=8). Points represent the average number of ecological momentary assessment (EMA) responses within a week across participants, and error bars represent a 95% CI around the mean value. Each trajectory represents a specific type of EMA response (eg, “Mindfulness without Headspace”) as indicated in the legend.

We observed a change over time. During weeks 1 and 2, parents reported using the Headspace app to support mindfulness activities. However, in weeks 3 and 4, mindfulness activities without the app increased (Figure 7).

**Table 2:** Self-reported engagement with any mindfulness activity across all users (n=8).

Parameters	No prompt	Prompt	Total
Ecological momentary assessments collected, n (%)	103 (62.4)	62 (37.6)	165 (100)
Engagements with any mindfulness behavior (with or without the app) indicated by parent report, n (%)	65 (63.1)	40 (65)	105 (100)
Engagements with mindfulness activity using the Headspace app indicated by parent report, n (%)	33 (32)	31 (50)	64 (100)

##### Emotional State or Affect

Across the 8 participants, the top three emotional states captured from the PAM responses were tired, satisfied, and sleepy. We did not observe any patterns or trends in emotional state over the course of the study, nor did we observe any trends between affect and engagement.

#### Acceptability

Acceptability and parent satisfaction were determined from free-response text on baseline and exit surveys, as well as from communication with parents (eg, during onboarding). Although 2 participants were affected by technology errors impacting EMA collection, they were included in exit surveys (n=10) as their experience could provide additional insight into outcomes. Overall, only 70% (7/10) found the program useful in managing stress, which is below our satisfaction benchmark of 90%. However, 90% (9/10) found the research helpful and would also recommend the program and Headspace app to a friend.

Specifically, most participants liked the wording of the message prompt (9/10, 90%) yet reported dismissing the prompt at some point over the course of the study (8/10, 80%). When specifically asked about the exit survey, 50% (5/10) indicated that the messages could have been more caring (eg, “We care about your health, take a min to manage your stress”) or humorous (“funnier”), but not authoritative (“do this now” type of message; 1/10, 10%). The majority (7/10, 70%) did not believe a visual within the prompt (eg, emoji or meme) would be helpful. Participants most often viewed prompts as reminders (9/10, 90%), while some participants (4/10, 40%) reported that prompts became “more annoying the longer I was in the study.” Just over half of the participants reported that both the timing and frequency of prompts were helpful (6/10, 60%). However, responses to open-ended questions highlighted the competing demands for parent attention in the moment:

I got a notification while I was out running errands.

P9

It felt like one more to-do on a long list of to-dos. I occasionally wanted to do mindfulness out and about while waiting for something, but I couldn't download them for offline use.

P3

Messages that were trying to be nice felt very tone deaf when circumstances conflicted with them, ie “the stressful part of your day is done” popping up on my phone as my baby is screaming.

P4

I had tons of other notifications and I would just delete them all at the same time.

P7

All participants (10/10, 100%) reported that the Headspace app was easy to use. While the majority (9/10, 90%) liked the *minimalistic and cuteness* of the graphics, 1 parent expressed a strong dislike of the narrator’s voice:

They’re simple and kind of cute.

P10

They were fun and pretty generic, kind of cute.

P7

The voice of the narrator. Not relaxing to be told what to do by a white dude.

P8

None of the activities emerged as most or least liked. Half of the participants were aware that Headspace had activities for children. While 40% (4/10) thought that using the app would help their child with stress management, only 1 (10%) parent tried the app with their 3-year-old child and reported that it was not a good experience. At the end of the study, 50% (5/10) reported decreases in stress, 40% (4/10) reported no change in stress levels, and 10% (1/10) reported an increase in stress levels:

At the end of the study, I found out I can do mindfulness exercises on my own without the app. The app taught me some tricks which I use a lot.

P10

## Discussion

### Principal Findings

We conducted a feasibility and acceptability study to begin to better understand when, how, and under what conditions a digital health intervention could support parental stress management in real time. The findings of this research demonstrate several areas for refinement before conducting a full-scale efficacy trial. First, in contrast to traditional mindfulness intervention recruitment efforts through mental health centers or community or school settings [20], we were able to meet our screening benchmark solely through efforts taken on the web and word of mouth, which did not include paid web-based advertisements (eg, Facebook advertisements). However, we lost nearly 67% (32/48) of eligible participants during enrollment, just shy of our enrollment benchmark (n=16; the benchmark was 20). This conversion rate is consistent with in-person, family-based health promotion programs, despite our intervention being offered without in-person or telehealth meetings. Parent interest in practical solutions for stress management was reaffirmed by the number of participants screened and eligible. However, subpar enrollment and onboarding (n=10; the benchmark was 16 or 100%) rates highlighted competing demands for parent attention, the need for simplicity in everyday solutions, and the ability to integrate into family routines. For those who completed onboarding, the 30-day intervention delivery was viewed as a success, based on 8 of 10 (80%) participants completing the intervention as intended (the benchmark was 80%) and the 72% EMA response rate (the benchmark was 60%). However, it is important to note that this subset may reflect parents who were highly motivated and technically savvy and may not be generalizable to a larger sample. Furthermore, our sample was predominantly mothers, similar to prior evidence [20], and lacked racial or ethnic diversity (the benchmark was 24%). Recruitment efforts may be strengthened by diversifying recruitment strategies to include both free and paid web-based advertisements, as well as traditional partnerships with the community, schools, and clinics.

Another limitation of the feasibility trial was technology acceptance. Familiarity with the Google Play Store and Headspace brand recognition facilitated onboarding and were viewed as helpful among this sample of parents. While leveraging commercial brands and academic-industry partnerships in research may be beneficial, it is not always possible. Owing to other overwhelming requests for partnership, Headspace was unable to commit to a formal partnership at the time of our request. The formative nature of the proposed work, limited research budget (eg, startup funds), and timing (desire to align the project implementation with the school calendar when family routines are more stable) likely limited any additional opportunities for partnership. Therefore, we used research-developed apps to capture smartphone paradata that would otherwise be proprietary to gain insight into real-world settings. In addition to passively capturing when and for how long the Headspace app was open, App Logger provided objective smartphone paradata that may have been otherwise overlooked, such as if a smartphone was set to silent when a prompt was delivered. However, technology acceptance of research-developed apps was impacted by concerns about privacy and familiarity, similar to other research [73,74]. The most notable challenge during onboarding was downloading an app from outside the Google Play Store (eg, GitHub). Unfamiliarity with installing apps from a source other than the Google Play Store contributed to inquiries from 50% (8/16) of the enrolled parents. Reliance on only Android devices is a common limitation of many mobile health studies that use open science research-developed apps. The Android operating system allows third-party apps to sample from more sensors and system smartphone logs than apps running on the Apple operating system. Efficient and cost-effective solutions in cross-platform mobile development are needed by industry (eg, Google and Apple) to help streamline resources available to researchers [75,76]. Conversely, in lieu of familiarity or brand recognition, family health researchers need to identify strategies for data collection that reduce participant burden (eg, passive data collection) but are viewed as acceptable.

Owing to variability in the literature, no benchmarks have been established for prompt verbiage, timing, or frequency. We deemed prompt wording acceptable in this population (9/10, 90%). However, both prompt frequency and timing were deemed unacceptable owing to subpar ratings (6/10, 60%), despite parents having control over prompt timing and prompt frequency subject to randomization. In exit surveys, most parents indicated that the prompts came at inconvenient times and were often viewed as reminders. Most dismissed the prompt at least once over 30 days (8/10, 80%). If a prompt was dismissed, most parents reported that it was because they were too busy. Only 1 of 10 (10%) parents reported that the study prompt was one of the *tons of other notifications* and would just delete them all. We found a lag of approximately 185 minutes from prompt delivery to action (eg, either launch Headspace app or dismiss notifications), which is congruent with other studies that found a delay of approximately 163 minutes between notification and action [51]. We observed the time-of-day variability for when the Headspace app was opened, noting that some participants tended to use the Headspace app around the same time each day, more so than others. However, there was insufficient data to investigate whether participants habituated to using the Headspace app during a particular time or in response to a prompt. This hypothesis is worth investigating in future trials to further inform habit formation. The literature is mixed on whether it is beneficial to give participants control over when a prompt is delivered. Prior research shows that both user-designated times to receive a prompt and giving the user control of prompt timing failed to enhance the use of a smartphone stress management app [77]. Intelligent prompts guided by sensor-driven machine learning algorithms that adapt to the user’s context may be beneficial for increasing user engagement [77-79]. It has also been suggested that tools to unobtrusively gauge and manage day-to-day stress may be improved by considering contextual information [34,40,80]. However, other research has shown that after 20 days of receiving machine learning suggestions, participants favored self-selecting their intervention, potentially seeking novelty after the algorithm became too locked in or limited in offerings [78]. However, little has been done to understand the nuanced context of everyday family life. We found that while most parents reported using a calendar to facilitate work and family schedules, family calendars captured the exceptions to everyday family routines (eg, appointments or practice) but did not account for daily family activities such as dinnertime, laundry, homework, crying babies, etc. A needs assessment conducted with a larger, more diverse sample and within the context of everyday family life may help to not only determine opportunities for stress support but may also help to identify how much support is needed based on stress severity.

Across all participants, we observed collectively higher engagement with the app on days when a prompt was sent and for a longer duration compared with days in which a prompt was not sent. However, engagement with apps decreased over time. This parallels other research suggesting that prompts impact near-time engagement with the app [30,77] but are not sustained over time [29,43,77,78]. We did not observe a descriptively higher engagement with any mindfulness activity on days when a prompt was sent. We observed more instances in which mindfulness activities were supported by the app versus without the app. Unfortunately, the Beehive app was only able to timestamp when a prompt was sent and unable to capture and record participant-indicated time frames for when to receive the prompt. On the basis of when prompts were sent, we observed peaks during the late morning and night hours. These findings are consistent with findings from a recent review of objective user engagement with 93 mental health apps [81]. Baumel et al [81] found that mindfulness apps were among the most used, with patterns of use exhibiting two peaks, in the morning (7 AM-9 AM) and at night (10 PM-midnight). We also observed that engagement with Headspace was higher in weeks 1 and 2 than in weeks 3 and 4. By weeks 3 and 4, we observed a potential trade-off, where trends in both self-reported and passively collected data indicated a decrease in app usage, while parent-reported mindfulness without the app increased. These findings suggest that mHealth interventions may help parents to learn or practice stress-regulating skills, to be further practiced or applied independent of the app.

In this study, we objectively observed app usage and self-reported interactions with the Headspace app. Most observed interactions with Headspace were ≤60 seconds in duration, while self-reported interactions averaged 5 to 10 minutes per day. This apparent discrepancy between the observed and reported values may reflect a few different scenarios. Unfortunately, a third EMA option to report using both strategies (mindfulness with the app and on their own) was overlooked during development, which may have contributed to some of the discrepancies. Findings may show a response bias, in which parents responded in a manner perceived as desirable. Alternatively, the discrepancy may reflect parents’ perceived time actually spent engaging with the app, such as on days when a prompt was sent. Finally, the prompts were capable of launching the Headspace app. Observations may reflect instances where parents intended to dismiss the prompt but accidentally launched and then closed the app. However, this finding is not necessarily a discrepancy and might reflect the self-reported aggregation of interim engagements throughout the day.

The findings suggest that parents may benefit from flexible interventions, allowing freedom to learn the necessary skills and practice or apply them during more resource-limited times. This is further supported by our findings that although app usage decreased over time, the use of learned skills increased over time. On the basis of this observation, disengagement with the app is not necessarily a negative finding. As suggested in the literature, sufficient versus sustained engagement may be a more useful gauge of engagement [34,41]. More research is needed to understand how much exposure to intervention content is necessary to support stress regulation. Despite the brief duration of app engagement, parents in this study reported that Headspace was easy to use and helpful in managing stress. Although the evidence favors a reduction in negative affect following a mindfulness intervention [22,82,83], we did not observe any patterns or trends. In a pilot study by Champion et al [26], participants who used Headspace showed improvements in self-reported life satisfaction, resilience, and perceived stress after 10 days, with medium to large effect sizes found in self-reported life satisfaction, resilience, and perceived stress after 30 days of use. However, participants in that study reported engaging with the app for at least 10 minutes per session and averaged 6.2 days use out of the first 10 days [26]. Other mHealth stress research examined the effects of microinterventions (eg, using a text prompt that instructs the user what to do along with a link to support the skill) [78]. They found benefits for interventions lasting >60 seconds, but those benefits diminished with usage time greater than approximately 200 seconds [78]. Morrison et al [77] found the average participant log-in to a smartphone stress app to be 4 minutes (240 seconds). They concluded that the timing and frequency of push notifications may increase the exposure to intervention content, but not necessarily use [77]. Prior research suggests that a few days of quick log-ins may be sufficient to enable users to learn new skills to practice without continued guidance from the app [41,47,77]. Breathing exercise apps have one of the lowest 30-day retention rates relative to apps that incorporate mindfulness, trackers, and peer support [81]. However, mindfulness apps often design guided meditations for repeated use [81]. Headspace activities favored by parents in this study focused on breathing, tips on how to make mindfulness a part of their day, and restlessness and sleep. Another strategy to consider in future work is to use a stealth health approach, where the intervention is woven into existing routines and the target (eg, stress management) is a side effect but not a primary motivator for participation [47,84].

### Limitations and Future Directions

This was a pilot MRT feasibility study designed to address the following question: *Can it work?* [58] Despite a myriad of recruitment strategies, it was challenging to recruit parents. Participants self-identified as parents who experienced stress and were interested in learning more about how mindfulness might help manage stress. Parenting stress, as evidenced during the pandemic, comes with unique challenges that differentiate this population from the general public. Our sample was predominantly White and female. This is similar to data on the characteristics of paid subscribers to the Calm app, another consumer-based mindfulness app [27]. Calm app users are also predominantly female (8778/10,981, 79.94%). It was difficult to recruit men or fathers compared with women or mothers and diverse populations, which is similar to several other family-based studies [47,85,86]. However, we strongly recommend conducting a needs assessment among diverse groups in future research efforts to include fathers, low-income populations, and racial or ethnic minorities who are often underrepresented in this literature. Finally, more research is warranted among pediatric populations, given the interrelated health between the parent and child. We found that parents did not use or find the Headspace kid pack useful. This differs from recent findings among a sample of parents who use the Calm app [87]. Just over half of the parents who use Calm (1537/2944, 52.21%) reported that older children used Calm to reduce stress, whereas younger children used Calm to improve sleep. A recent review of preventive digital mental health interventions for children and young people (n=21 interventions) found a need for more (1) research among children aged ≤10 years, (2) co-design processes with children, and (3) research among children from vulnerable and underserved backgrounds [88].

### Conclusions

Preliminary findings suggest that parents are receptive to using mindfulness apps to support stress management, and prompts are likely to increase engagement with the app. However, we identified several implementation challenges in the current trial, specifically the need to optimize prompt timing and frequency as a strategy to engage users in preventive digital health. Commercially available mindfulness apps appear acceptable among this sample of parents and may provide an opportunity to expose parents to mindfulness skills that may be later practiced without the app. More research is warranted to understand how much time is necessary to spend using a stress management app for parents to learn the necessary skills and translate those skills into everyday life. As the field of mHealth evolves, strategies to engage users in preventive digital mental health interventions must also evolve to increase the likelihood of intervention success.

## Appendix

Multimedia Appendix 1 Descriptive demographics.

Multimedia Appendix 2 Push notification text.

## Abbreviations

DASS-21: Depression Anxiety Stress Scale 21-item
EMA: ecological momentary assessment
IRB: Institutional Review Board
JITAI: just-in-time adaptive intervention
mHealth: mobile health
MRT: microrandomized trial
PAM: Photographic Affect Meter
PI: principal investigator
