# Supplementary material for: Digital Prompts to Increase Engagement With the Headspace App and for Stress Regulation Among Parents: Feasibility Study
Source: JMIR Form Res. 2022 Mar 21;6(3):e30606. doi: 10.2196/30606 (PMC8981020; doi:10.2196/30606)
Supplement: Multimedia Appendix 1 [file formative_v6i3e30606_app1.docx]

## Web Appendix 1

Table 1. Descriptive characteristics of participants at baseline (n=16).

| Variable | Range | N (%) | |
| --- | --- | --- | --- |
| Age (years) | 18-24 | 1 | 6.25 |
|  | 25-34 | 5 | 31.25 |
|  | 35-44 | 9 | 56.25 |
|  | 45+ | 1 | 6.25 |
| Gender | Female | 14 | 87.5 |
|  | Male | 2 | 12.5 |
| Race | Caucasian | 15 | 93.75 |
|  | African American | 1 | 6.25 |
| Education | Some college | 3 | 18.75 |
|  | 2-year degree (Associate) | 1 | 6.25 |
|  | 4-year degree (Bachelors) | 3 | 18.75 |
|  | Masters degree | 6 | 37.5 |
|  | Doctoral degree | 3 | 18.75 |
| Public assistance | No | 14 | 87.5 |
|  | Yes | 2 | 12.5 |
| Relationship status | Married | 14 | 87.5 |
|  | Separated or divorced | 2 | 12.5 |
| Number of children living at home | One | 8 | 50 |
|  | Two | 7 | 43.75 |
|  | Three | 1 | 6.25 |
| Depression | Normal | 15 | 93.75 |
|  | Mild | 0 | 0 |
|  | Moderate | 1 | 6.25 |
|  | Severe | 0 | 0 |
|  | Extremely severe | 0 | 0 |
| Anxiety | Normal | 14 | 87.5 |
|  | Mild | 1 | 6.25 |
|  | Moderate | 1 | 6.25 |
|  | Severe | 0 | 0 |
|  | Extremely severe | 0 | 0 |
| Stress | Normal | 15 | 93.75 |
|  | Mild | 1 | 6.25 |
|  | Moderate | 0 | 0 |
|  | Severe | 0 | 0 |
|  | Extremely severe | 0 | 0 |
| Do you typically practice managing your stress? | Yes | 13 | 81.25 |
|  | No | 3 | 18.75 |
| Have you ever used mindfulness in the past? | Yes | 6 | 37.5 |
|  | No | 10 | 62.5 |
| Have you ever used the Headspace app in the past? | Yes | 1 | 6.25 |
|  | No | 15 | 93.75 |
