# Supplementary material for: Digital Prompts to Increase Engagement With the Headspace App and for Stress Regulation Among Parents: Feasibility Study
Source: JMIR Form Res. 2022 Mar 21;6(3):e30606. doi: 10.2196/30606 (PMC8981020; doi:10.2196/30606)
Supplement: Multimedia Appendix 2 [file formative_v6i3e30606_app2.docx]

## Web Appendix 2

List of push notifications. Push notification text was randomized from the list below:

1. The stressful part of your day is over.

2. You deserve 10 minutes of headspace.

3. Take 10.

4. Decompress, give your mind some rest.

5. Take 10. Rejuvenate your mind.

6. Headspace is your calm place. Take 10.

7. Take your mind to a/your calm place.

8. Get some headspace.

9. Make the next 10 minutes yours. You deserve them.

10. Need space? Take 10.

11. 10 minutes of space is all you need

12. It takes 10 to get your space

13. 10 minutes for yourself can make a huge difference

14. Make space in your head

15. Give your mind a break

16. Now is the time; take 10

17. You deserve headspace

18. Practice mindfulness by noticing how you feel doing different things

19. Create a sense of ease and be present

20. Use visualization to recharge your mind and body

21. Practice gentle focus to bring yourself to a state of calm

22. Take time to be alone for a few and allow yourself to unwind

23. Let go of the past to enjoy the present

24. Let go of tension and find peace

25. Give yourself time to recharge

26. Move towards a calmer mindset

27. Find the spot between focus and relaxation

28. Be in the moment to connect with those around you

29. Use mindfulness to develop your mental toughness

30. Take a break and let go of distractions
